# Perioperative Management of a Hip Fracture Complicated With Acute Colonic Pseudo-Obstruction (Ogilvie's Syndrome): A Case Report

**DOI:** 10.7759/cureus.86926

**Published:** 2025-06-28

**Authors:** Hamza Zemrani, Larbi Aberouch, Salim Chajai, Jawad Tadili, Mohammed Ali Ech-cherif el Kettani, Mamoun Faroudy

**Affiliations:** 1 Surgical Intensive Care Unit, Ibn Sina University Hospital, Rabat, MAR; 2 Anesthesiology and Critical Care, Ibn Sina University Hospital, Rabat, MAR; 3 Emergency Surgery Critical Care, Université Mohammed V Rabat, Rabat, MAR; 4 Anesthesia and Critical Care, Ibn Sina University Hospital, Rabat, MAR; 5 Anesthesia and Critical Care, Moulay Youssef Hospital, Rabat, MAR

**Keywords:** acute colonic pseudo-obstruction, geriatric anesthesia, geriatric hip fracture, ogilvie's syndrome, perioperative management, trauma anesthesia

## Abstract

Acute colonic pseudo-obstruction (Ogilvie syndrome) is characterized by a marked dilation of the colon in the absence of mechanical obstruction. This rare syndrome can occur in at-risk patients after trauma and in the preoperative period. We report on the case of a patient who presented with acute colonic pseudo-obstruction after a hip fracture, who was treated conservatively for her Ogilvie syndrome and then operated on for the fracture. This report highlights the perioperative challenges related to this condition and the importance of a timely diagnosis and management.

## Introduction

Acute colonic pseudo-obstruction (ACPO), or Ogilvie’s syndrome, is a form of colonic dilation without an underlying mechanical or anatomical cause. The dilation primarily affects the cecum and the ascending colon. This condition most commonly occurs in elderly adults with multiple comorbidities, but it can also develop in otherwise healthy individuals in the context of trauma or surgical intervention [1].

In the post-traumatic and orthopedic setting, the incidence of ACPO ranges from 0.3% to 3% [2,3], particularly following trauma or surgeries involving the pelvis, hip, or knee.

ACPO is considered complicated when a patient shows signs of intestinal ischemia, peritonitis, or perforation; the risk of complications increases directly with the diameter of the cecum and the duration of the disease [4].

Many patients recover with early and appropriate management, whereas morbidity and mortality increase in those who develop complications [4,5]. We report the case of a 75-year-old woman who developed ACPO following a hip fracture, for which surgical intervention was planned. The perioperative setting adds additional challenges to the management of this condition.

## Case presentation

We report the case of a 75-year-old woman with a medical history of arterial hypertension treated with losartan, atrial fibrillation treated with rivaroxaban, and Alzheimer’s disease treated with memantine and quetiapine. The patient also had a hysterectomy eight years prior for endometrial cancer. The patient was otherwise able to ambulate independently and lived in a retirement facility.

**Figure 1:**
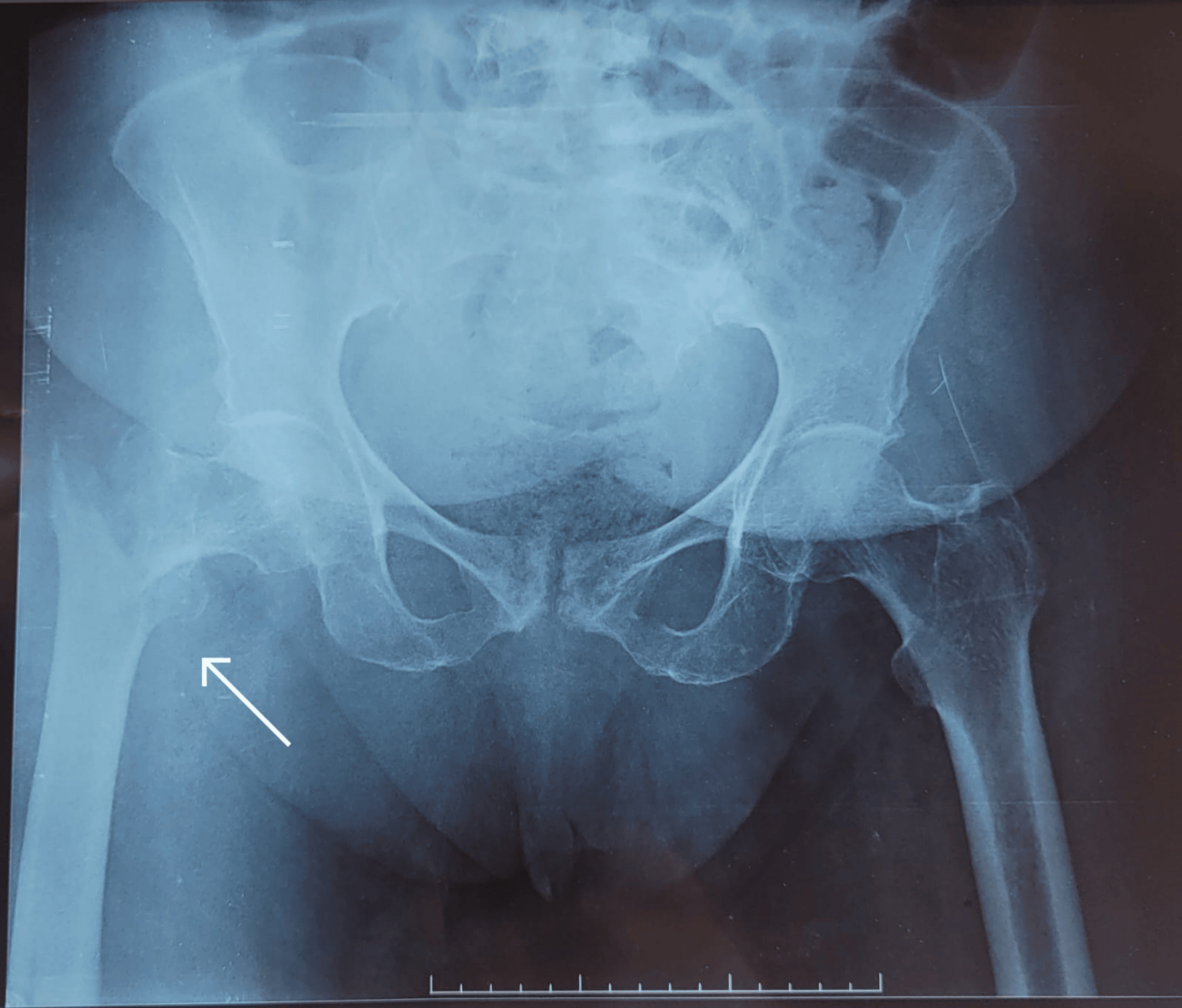
Right intertrochanteric fracture

The patient suffered a fall three days before admission to the intensive care department, resulting in a right intertrochanteric femoral fracture (Figure 1). The day after the fall, she presented with vomiting, constipation, abdominal pain, and distension. On physical examination, the patient was conscious, with an irregular heart rate of 140 beats per minute, and arterial blood pressure was at 160/80 mmHg. Her respiratory rate was at 20 cycles per minute and her oxygen saturation was at 94% on room air. The abdomen was distended and tympanic on percussion. The patient was admitted to the intensive care department for preoperative evaluation and management.

Laboratory tests revealed hemoglobin at 10 g/dL, serum potassium at 3.29 mmol/L, and blood glucose at 2.2 g/L with renal failure with urea at 0.83 g/L and creatinine at 15.6 mg/L; her arterial lactate level was at 0.9 mmol/L and HCO3- was at 20.4 mmol/L (Table 1).

**Table 1:** Laboratory test results

Laboratory test	Result	Reference values
Hemoglobin	10 g/dl	12 - 16 g/dl
Serum potassium	3.29 mmol/L	3.5 – 5.5 mmol/L
Blood glucose	2.2 g/L	0.8 - 1 g/L
Urea	0.83 g/L	0.13 – 0.43 g/L
Creatinine	15.6 mg/L	5 - 13 mg/L
Arterial lactate	0.9 mmol/L	<2 mmol/L
HCO3-	20.4 mmol/L	22 - 29 mmol/L

**Figure 2:**
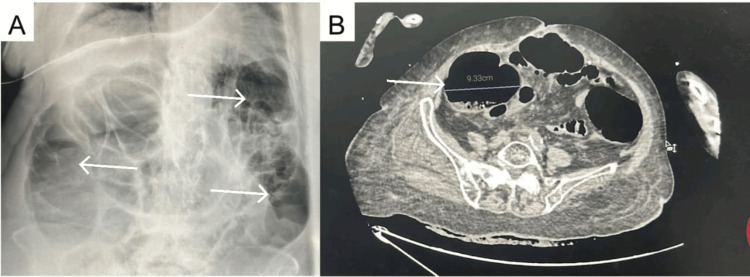
A. Plain abdominal radiograph showing an air-distended colon (arrow) B. CT scan showing a cecal diameter of 9.3 cm (arrow)

A CT scan (Figure 2) was performed without injection because of the renal failure, showing a diffuse dilation of the large bowel without a transition point to suggest obstruction, with a cecal diameter of 9.33 cm and a rectal fecaloma. The ECG showed atrial fibrillation at 135 beats per minute. Chest X-ray revealed bilateral basal atelectasis. Transthoracic echocardiography showed a preserved ejection fraction at 55%, a non-dilated right ventricle, and an intermediate probability of pulmonary hypertension. Lower limb venous ultrasound ruled out thrombosis. After the exclusion of a mechanical cause, a diagnosis of acute colonic pseudo-obstruction (Ogilvie’s syndrome) was made.

Conservative management was initiated using nasogastric tube stomach decompression, metoclopramide, rectal enemas, and digital evacuation for the fecaloma and bowel rest with parenteral nutrition using a triple-chamber, all-in-one parenteral solution at a dose of 24 kcal/kg/day. Hypokalemia was corrected with intravenous potassium, hypovolemia with cautious fluid replacement, and atrial fibrillation was managed using intravenous amiodarone, considering that the patient was already anticoagulated. Non-opioid analgesia (acetaminophen) was provided, and glycemia was corrected using insulin.

**Figure 3:**
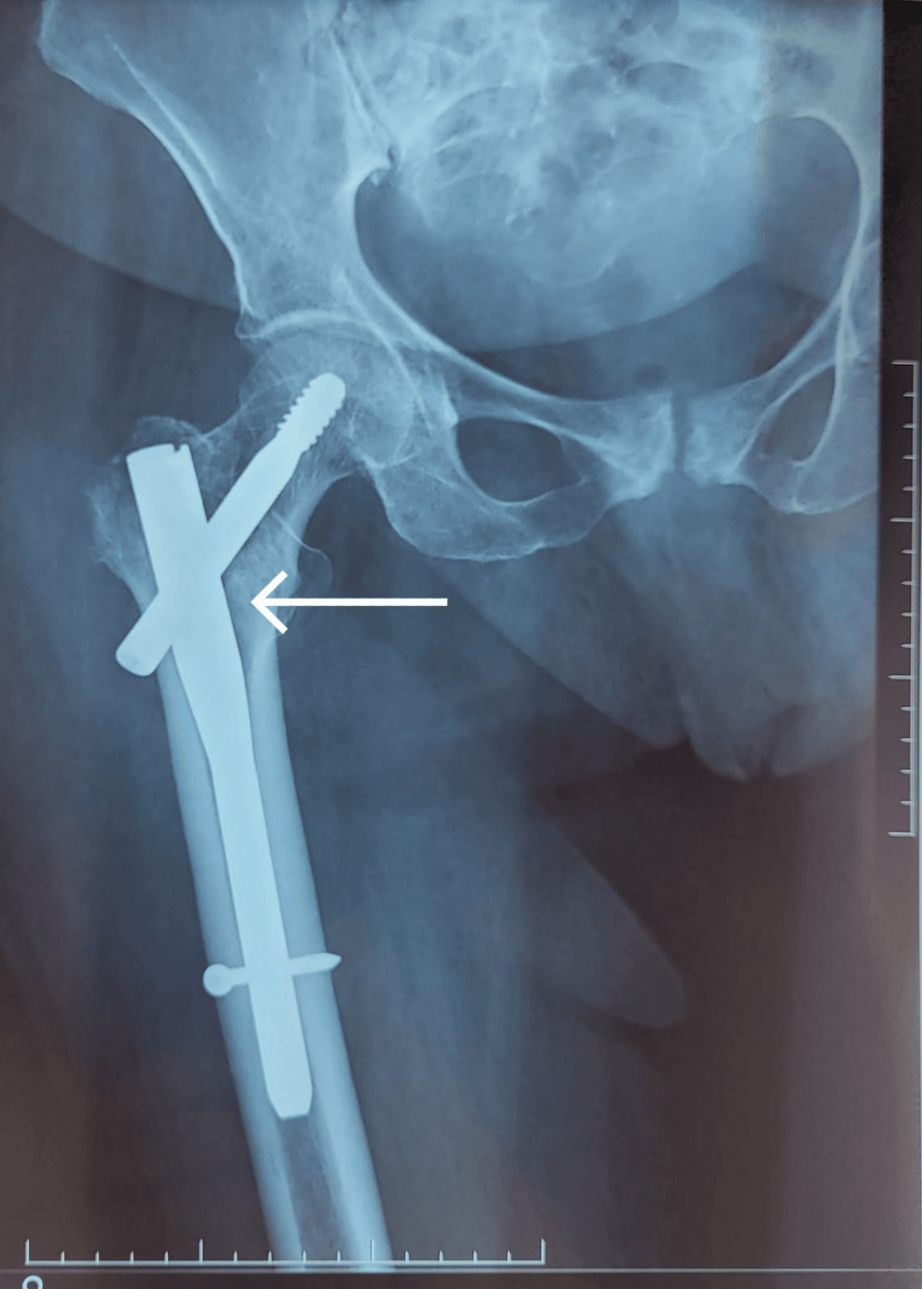
Postoperative radiograph showing the gamma nail (arrow)

On the third day in the intensive care, fecal evacuation was achieved, the vomiting subsided, and abdominal distension improved. The intertrochanteric fracture was surgically treated with a gamma nail under general anesthesia (Figure 3). Postoperatively, the patient required mechanical ventilation for three days after the surgery due to atelectasis and residual sedation. She was extubated following lung recruitment maneuvers, blood transfusion, and fluid optimization. The patient spent three additional days in the intensive care unit receiving noninvasive ventilation. She was weaned from oxygen, presented good respiratory mechanics, normalized renal function, resumed oral feeding, and then was transferred to the orthopedic surgery department. She presented no confusion or delirium during her stay.

## Discussion

Acute colonic pseudo-obstruction, also called Ogilvie’s syndrome, is characterized by a marked dilation of the colon in the absence of mechanical obstruction [6]. In a review of 400 cases of acute colonic pseudo-obstruction, the most commonly associated condition was nonoperative trauma, mostly extremity fractures. Surgical procedures most commonly associated with the syndrome were cesarean section and hip surgery, with an onset at an average of 4.5 postoperative days. Associated medical conditions included cardiac diseases, infections, and neurological problems [1].

The typical patient with pseudo-obstruction is infirm, unwell, and has multiple comorbidities [7]. Our patient had many risk factors for Ogilvie’s syndrome adding to her hip fracture, including Alzheimer’s disease and taking antipsychotic medications. Indeed, quetiapine has anticholinergic properties and may, with memantine, have additive inhibitory effects on colonic mobility. In a database of 102 cases of suspected life-threatening clozapine-induced gastrointestinal hypomotility, there was a mortality rate of 27.5% and considerable morbidity. The mechanism was likely to be anticholinergic and antiserotonergic [8].

The pathophysiology of acute colonic pseudo-obstruction is incompletely understood. Ogilvie initially theorized that unopposed parasympathetic activity led to excessive and uncoordinated muscle contraction; the intestine does not relax, leading to pseudo-obstruction. More recent hypotheses include an excess of sympathetic (inhibitory) motor input to the intestines, decreased parasympathetic (excitatory) motor input, and excess stimulation of peripheral µ opioid receptors by endogenous or exogenous opioids [9]. A role for the colocolonic reflex, mediated by sympathetic nerves through α-2 adrenergic receptors, was suggested, reinforcing the sympathetic excess hypothesis [7,10].

A timely diagnosis is of paramount importance to prevent complications and decrease mortality. An increase in the colonic diameter, according to Laplace’s law, increases tension in the colonic wall, increasing the risk of perforation and ischemia. The risk of colonic perforation is greater with cecal diameters higher than 12 cm and when the dilation has been lasting longer than six days. Acute colonic pseudo-obstruction diagnosis remains one of exclusion; our patient presented with abdominal distension, vomiting, and constipation, and there was no evidence of mechanical obstruction on the CT scan. A contrast-enhanced CT may have been useful to rule out ischemia, but we chose not to inject contrast given the presence of acute kidney injury in this frail patient and because her general condition wasn’t consistent with intestinal ischemia. The cecal diameter was at 9.33 cm, which warranted a conservative treatment as recommended when the cecal diameter was lower than 12 cm [4].

Early hip fracture surgery (within one to two days from admission) is associated with a lower risk of death and pressure sores [11]; however, in the context of acute colonic pseudo-obstruction, some preoperative optimization may be warranted. In our patient, Ogilvie syndrome caused vomiting with hypovolemia, hypokalemia, and acute kidney injury. The abdominal distension and increased intra-abdominal pressure may have contributed to the kidney injury and to a restrictive syndrome with atelectasis. The hypokalemia accompanying acute colonic pseudo-obstruction can be a consequence of potassium loss through vomiting, as can the colonic hypomotility be worsened or maintained by the hypokalemia. Symptomatic treatment with fluid resuscitation and electrolyte replacement is essential in this setting.

The conservative management of patients with acute colonic pseudo-obstruction includes maintaining nil oral intake, nasogastric tube decompression, rectal tube insertion, cessation of offending medications, position changing, and neostigmine when the cecal diameter is higher than 12 cm [7]. We did not use neostigmine in our patient because she was already on amiodarone for her atrial fibrillation, and there was a concern for developing bradycardia, but we used metoclopramide, though it showed inconsistent results in case reports [12,13]. The conservative management proved to be efficient, as the patient resumed her intestinal transit on the third day in the intensive care unit, and with the improvement in her abdominal distension and correction of hydroelectrolytic anomalies, she was considered fit for surgery.

In the setting of acute colonic pseudo-obstruction, a few case reports support the use of spinal anesthesia at a level of T4 to relieve the obstruction, and one study in 18 patients found low-dose epidural anesthesia to be safe and effective in the treatment of Ogilvie syndrome, the mechanism being sympathetic denervation to the intestine and increased propulsive activity of the bowel [14-16]. It is difficult to assert a direct causal link between the anesthesia technique and relief of the obstruction; however, prolonged analgesia with regional anesthesia techniques and lower use of opioids may also be beneficial in this setting. We opted for general anesthesia because of the respiratory status of our patient, as she may not have tolerated a supine position with a potential respiratory muscle blockade.

In a retrospective cohort of 1060 patients with pelvic and/or acetabular fractures, 25 patients were diagnosed with Ogilvie syndrome perioperatively, and it was associated with a significant increase in nosocomial infections, sepsis, pulmonary embolism, ICU stay, and prolonged hospital admission [2]. Our patient had a prolonged intensive care unit stay and hospital admission because of ongoing respiratory management and residual sedation. The situation may have been worse in the absence of a timely diagnosis and conservative management, and this highlights the importance of raising awareness about this syndrome in the traumatic and preoperative context in populations at risk.

## Conclusions

Ogilvie syndrome is a diagnosis of exclusion that should be considered in at-risk patients following trauma and in the preoperative setting. Timely diagnosis allows for more conservative management and avoids complications and mortality. The anesthesia technique depends on the clinical presentation, and the physiological effects of anesthesia on intestinal motility should be accounted for.
